# Adapting for life in the extreme

**DOI:** 10.7554/eLife.48999

**Published:** 2019-07-15

**Authors:** Carolin M Kobras, Daniel Falush

**Affiliations:** 1 Milner Centre for EvolutionUniversity of BathBathUnited Kingdom

**Keywords:** horizontal gene transfer, lateral gene transfer, Cyanidiales, red algae, evolution, genome, Other

## Abstract

Red algae have adapted to extreme environments by acquiring genes from bacteria and archaea.

**Related research article** Rossoni AW, Price DC, Seger M, Lyska D, Lammers P, Bhattacharya D, Weber APM. 2019. The genomes of polyextremophilic Cyanidiales contain 1% horizontally transferred genes with diverse adaptive functions. *eLife* **8**:e45017. doi: 10.7554/eLife.45017

Most humans have nearly the same complement of genes, all of which have come from our primate ancestors (Salzberg, 2017). On the other hand, even closely related strains of the bacterium *Escherichia coli* can differ by hundreds of genes (Touchon et al., 2009) despite having a much smaller genome. These genes have been acquired via a process called horizontal gene transfer (HGT), which is an important driver of adaptation, as it allows bacteria and other prokaryotes to gain the genes they need in order to thrive in certain environments (Koonin et al., 2001). Moreover, this exchanging of genes has resulted in many genetic elements in prokaryotes becoming highly mobile, making it easier for DNA to be transferred to a diverse range of hosts.

HGT has also been observed in animals, plants and other eukaryotes (Husnik and McCutcheon, 2018), but its role in determining genome composition and facilitating adaptation in these species remains unclear (Ku and Martin, 2016). Now, in eLife, Andreas Weber and co-workers at Heinrich Heine University, Arizona State University and Rutgers University – including Alessandro Rossoni as first author – report evidence for HGT between prokaryotes and the red alga Cyanidiales (Rossoni et al., 2019). These are remarkable single-cell organisms that can perform photosynthesis at temperatures up to 56°C, and can live in extreme environments such as hot springs and acid rivers (Schönknecht et al., 2013). Cyanidiales can also be used to investigate HGT over geological timescales because they share a common ancestor that dates back 800 million years to a time before animals had even evolved.

Based on an analysis of ten new and three previously reported Cyanidiales genomes, Rossoni et al. found that 1% of genes had been obtained via HGT. Moreover, many of these genes coded for proteins that were needed to survive in extreme environments (such as proteins involved in detoxifying heavy metals like arsenic or mercury, or removing free radicals; Figure 1). Additionally, prokaryotes adapted to the same extreme environment as Cyanidiales were commonly identified as the source of these genes. It seems likely, therefore, that HGT influenced the evolution of Cyanidiales, especially because the criterion used to detect HGT was conservative and the study did not attempt to detect gene transfer from other eukaryotes.

**Figure 1.**
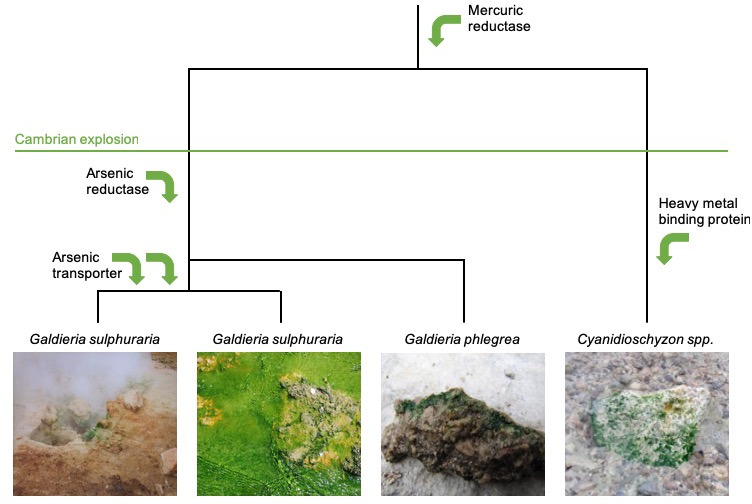
Horizontal gene transfer in the evolution of red algae. The evolutionary trajectory of the red algae Cyanidiales is shown from top to bottom. Rossini et al. investigated genetic changes that took place before and after the Cambrian explosion 541 million years ago, and found that Cyanidiales obtained 1% of their genes during this time by horizontal transfer. Many of these genes allowed Cyanidiales to adapt to extreme environments, such as genes related to the detoxification of heavy metals including mercury and arsenic (represented by green arrows). Some of the lineages of Cyanidiales that were sequenced by Rossoni et al. are shown in the bottom panels: two of these have the same taxonomic name despite having diverged from one another millions of years ago. Image credit: Andreas Weber (left panel), Debashish Bhattacharya (two middle panels), and Shin-ya Miyagishima (right panel).

Comparing the new Cyanidiales genes to genes found in present-day bacteria and archaea databases did not yield any recent examples of HGT. This absence of recent events is unsurprising, as Rossoni et al. estimated that Cyanidiales acquire just one gene via HGT every 14.6 million years – the same amount of time it took for humans to diverge from the orangutan. Such a low rate makes finding a fresh transfer in a small number of genomes unlikely. Instead, the majority of HGT candidate genes found by Rossoni et al. have acquired introns (non-protein coding segments of DNA), and then persisted over hundreds of millions of years.

Despite there being evidence to show HGT occurred, it still remains unclear how these transfers took place. The best-studied mechanisms by which eukaryotes acquire DNA from other organisms are sexual reproduction and by transferring DNA from symbionts (biological organisms that live cooperatively with other organisms). However, meiotic sex only occurs between closely related species, and therefore cannot explain how Cyanidiales appear to have gained DNA from such a diverse range of prokaryotes: moreover, the evolution of symbiotic transfer is uncommon in most taxonomic groups. Instead DNA was more likely obtained via viral infection or plasmids (circular molecules of double stranded DNA) being transferred between prokaryotes and eukaryotes (Heinemann and Sprague, 1989). Indeed, a recent study has shown that many eukaryotes, including red algae, can acquire plasmids carrying genes derived from plants, viruses and bacteria (Lee et al., 2016).

The work of Rossoni et al. suggests that, in terms of gene content evolution, Cyanidiales are more similar to humans than to *E. coli*, which is consistent with previous qualitive comparisons of HGT patterns in eukaryotes and prokaryotes (Ku and Martin, 2016). However, a number of mysteries still remain. For example, what are the most common modes of plasmid transmission in Cyanidiales? How do plasmids maintain themselves in populations? How often do they jump between species, and how far do they jump? To answer these questions we should first observe what is happening all around us today (Popa et al., 2017) and, if possible, study events that occur more frequently than once every 14.6 million years.
